# Maxillary lateral incisor agenesis is associated with maxillary form: a geometric morphometric analysis

**DOI:** 10.1007/s00784-022-04690-9

**Published:** 2022-08-29

**Authors:** Michael Nemec, Linda Schwarz, Michael H. Bertl, Kristina Bertl, André Gahleitner, Philipp Mitteroecker, Erwin Jonke

**Affiliations:** 1 Division of Orthodontics, University Clinic of Dentistry, Medical University of Vienna, 1090 Vienna, Austria; 2 Department of Orthodontics, Sigmund Freud University Vienna, 1020 Vienna, Austria; 3 Department of Periodontology, Faculty of Odontology, University of Malmö, Malmö, Sweden; 4 Division of Oral Surgery, University Clinic of Dentistry, Medical University of Vienna, Vienna, Austria; 5 Department of Diagnostic Radiology, Division of Osteoradiology, General Hospital, Medical University of Vienna, Vienna, Austria; 6 Department of Evolutionary Biology, Unit for Theoretical Biology, University of Vienna, 1030 Vienna, Austria

**Keywords:** Dental agenesis, Maxillary lateral incisors, Geometric morphometrics, Craniofacial morphology

## Abstract

**Background and objective:**

Agenesis of the maxillary lateral incisor occurs in up to 4% of all individuals and requires multidisciplinary treatment. Its developmental origins, however, are not fully understood. Earlier studies documented genetic factors contributing to agenesis but also an association with craniofacial morphology. In this study, we assessed the association between maxillary morphology and lateral incisor agenesis by a geometric morphometric approach to disentangle the roles of developmental plasticity and genetic factors.

**Materials and methods:**

We quantified the maxillary alveolar ridge by 19 two-dimensional landmarks on cross-sectional images of 101 computed tomography scans. We compared the shape and size of the alveolar ridge across patients with unilateral or bilateral agenesis of maxillary lateral incisors and patients with extracted or in situ incisors.

**Results:**

The maxillary alveolar ridge was clearly narrower in patients with agenesis or an extracted incisor compared to the control group, whereas the contralateral side of the unilateral agenesis had an intermediate width. Despite massive individual variation, the ventral curvature of the alveolar ridge was, on average, more pronounced in the bilateral agenesis group compared to unilateral agenesis and tooth extraction.

**Conclusions:**

This suggests that pleiotropic genetic and epigenetic factors influence both tooth development and cranial growth, but an inappropriately sized or shaped alveolar process may also inhibit normal formation or development of the tooth bud, thus leading to dental agenesis.

**Clinical relevance:**

Our results indicate that bilateral agenesis of the lateral incisor tends to be associated with a higher need of bone augmentation prior to implant placement than unilateral agenesis.

**Supplementary Information:**

The online version contains supplementary material available at 10.1007/s00784-022-04690-9.

## Introduction

After third molars and lower second premolars, maxillary lateral incisors are the third most common congenitally missing teeth [1, 2] with a reported prevalence of about 4% [3]. Maxillary bone is a complex structure, surrounded by muscular, dental, and skeletal components, which contribute to mastication, speech, and breathing [4]. Missing maxillary lateral incisors often pose a challenge to dentists and patients. There are four main treatment options for maxillary lateral incisor agenesis that typically require a multidisciplinary treatment approach: an implant-supported crown, a tooth-supported restoration, orthodontic space closure, or auto-transplantation [5–10]. An implant or transplanted root surface requires full bony coverage and, hence, a sufficiently sized alveolar process in this region. A sound understanding of maxillary form and the required alveolar characteristics are pivotal in the decision for the best treatment choice.

It has been shown that the anterior maxilla is prone to bone loss after tooth extractions in this area [11]. Resorptive activity during postnatal ontogeny in the anterior part of the maxilla has been described by various authors [12, 13]. Previous studies have suggested that the resorptive field within the anterior maxilla increases in size during ontogeny but also tooth extractions can lead to resorptive alveolar ridge remodeling. In an earlier radiographic study on patients with congenitally missing maxillary lateral incisors, it has been shown that mesio-distal gap width negatively correlates with edentulous alveolar ridge dimensions, i.e., an increased gap width was associated with a reduced alveolar ridge dimension [14].

While these studies clearly document an influence of tooth extraction or agenesis on maxillary form, the influence of maxillary size and shape on dental agenesis is less obvious. Various studies indicate that dental agenesis may not be purely genetically determined [15–17] because it is also associated with craniofacial morphology [18–21]. As tooth development proceeds concomitantly with alveolar growth, alveolar dimensions may influence odontogenesis [20, 22]: a too small or inappropriately shaped alveolar process may inhibit normal formation or development of the tooth bud, leading to agenesis of a tooth in this anatomical region. Beyond correlation studies, such an effect of skeletal dimensions on human tooth development cannot be directly assessed, but comparative and experimental studies in animals are in line with this explanation. Evolutionary trends of tooth loss in primates and other mammals show particular patterns and tend to occur in the inverse order as they develop ontogenetically (i.e., the first tooth to be lost is the one that develops last in a certain class of teeth, e.g., lateral incisor, third molar) [23–25]. An evolutionary decrease in the number of teeth in primates is also associated with reduced demands on mastication and the size of the jaw [26, 27]. Similarly, it has been suggested that the reduction of the number of teeth in modern humans has resulted from the shortening of the jaws [28–30]. Also among modern humans, craniofacial dimensions have been reported to correlate with third molar agenesis [31, 32].

However, correlations between human cranial dimensions and dental agenesis not necessarily result from unidirectional pathways only, i.e., from the effects of dental agenesis on bone remodeling and the effects of bone dimensions on tooth development. Certain genetic and epigenetic factors may additionally influence the development of both the cranium and the teeth. The fact that the major genetic drivers of tooth development, such as Shh and members of the Fgf, Wnt, and Bmp gene families, also determine craniofacial growth seems to support this “pleiotropy hypothesis” [33–35]. Such pleiotropic factors may also be reflected by the common co-occurrence of dental agenesis with other dental and craniofacial anomalies [36–38]. A morphometric study on mandibular form and second premolar agenesis also concluded that (epi) genetic factors with influences on both skeletal and dental development most likely account for the observed skeleto-dental associations [20].

Geometric morphometric analyses, which potentially allow disentangling the roles of developmental plasticity and genetic factors, are rarely used in dentistry in general and in the study of dental agenesis specifically. Altogether, to the best of our knowledge, developmental origins of lateral incisor agenesis are still not fully understood and need to be further investigated. Hence, in the present study, we assessed the association between maxillary morphology and lateral incisor agenesis by comparing the shape and size of maxillary cross-sections across adult patients with unilateral or bilateral agenesis of maxillary lateral incisors and patients with extracted or in situ incisors. Two-dimensional landmarks and semilandmarks were collected on cross-sections of CT scans and analyzed by geometric morphometric methods [39–41]. Based on these data, we aimed at testing three hypotheses [21]:

**H1**: Agenesis causes a change in maxillary morphology due to inadequate alveolar ridge development in the area of the missing tooth (maxillary plasticity).

**H2**: Agenesis is caused by spatial limitations within the maxilla (dental plasticity).

**H3**: Common genetic/epigenetic factors cause agenesis and affect maxillary form (pleiotropy).

## Material and methods

### Imaging

The retrospective study sample consisted of 101 CT scans of patients treated at the University Clinic of Dentistry, Medical University of Vienna (see [14] for more details). All CT scans were indicated by the treating dentist at that time due to medical reasons following the ALARA principle and not due to study purpose. CT scans were recorded following a standard dental CT investigation protocol [42] and a high-resolution bone algorithm with a Tomoscan SR 6000 (Philips Medical Systems, Eindhoven, the Netherlands; 75 mA, 120 kV, 2 s scanning time, 1 mm slice thickness, field of view [FoV]: 90 mm) or with a Somatom Sensation 4 (Siemens, Forchheim, Germany; 80 mA, 120 kV, 1 s scanning time, 0.5 mm slice thickness, FoV: 90 mm). Ortho-radial multiplanar reconstructions were calculated orthogonally to a manually drawn central line of the jaw arch in the axial plane.

Patients were divided into three groups: agenesis of a lateral incisor, edentulous alveolar ridge at least three months after extraction of a lateral incisor, and a control group with lateral incisors in situ. If lateral incisors were missing bilaterally, one side was chosen by coin toss, and the other side was considered the contralateral side. CT scans were screened for the following inclusion criteria in order to reduce the effects of potentially confounding factors: an age of 18 years or older at the time of the scan, presence of regularly erupted adjacent teeth, and a minimum mesio-distal gap dimension of 4 mm at the marginal bone level. Exclusion criteria were cleft lip and/or cleft palate, previous augmentation procedure, and a cystic lesion exceeding 5 mm in diameter at the time of tooth loss in the extraction group. All available CT scans between 2004 and 2014 were screened for theses eligibility criteria. Patients fulfilling the criteria were included consecutively until the intended sample size was reached. Demographic data such as patient age and sex as well as data on orthodontic treatment prior to CT scanning were acquired from the patients’ dental records.

### Measurements

**Fig. 1:**
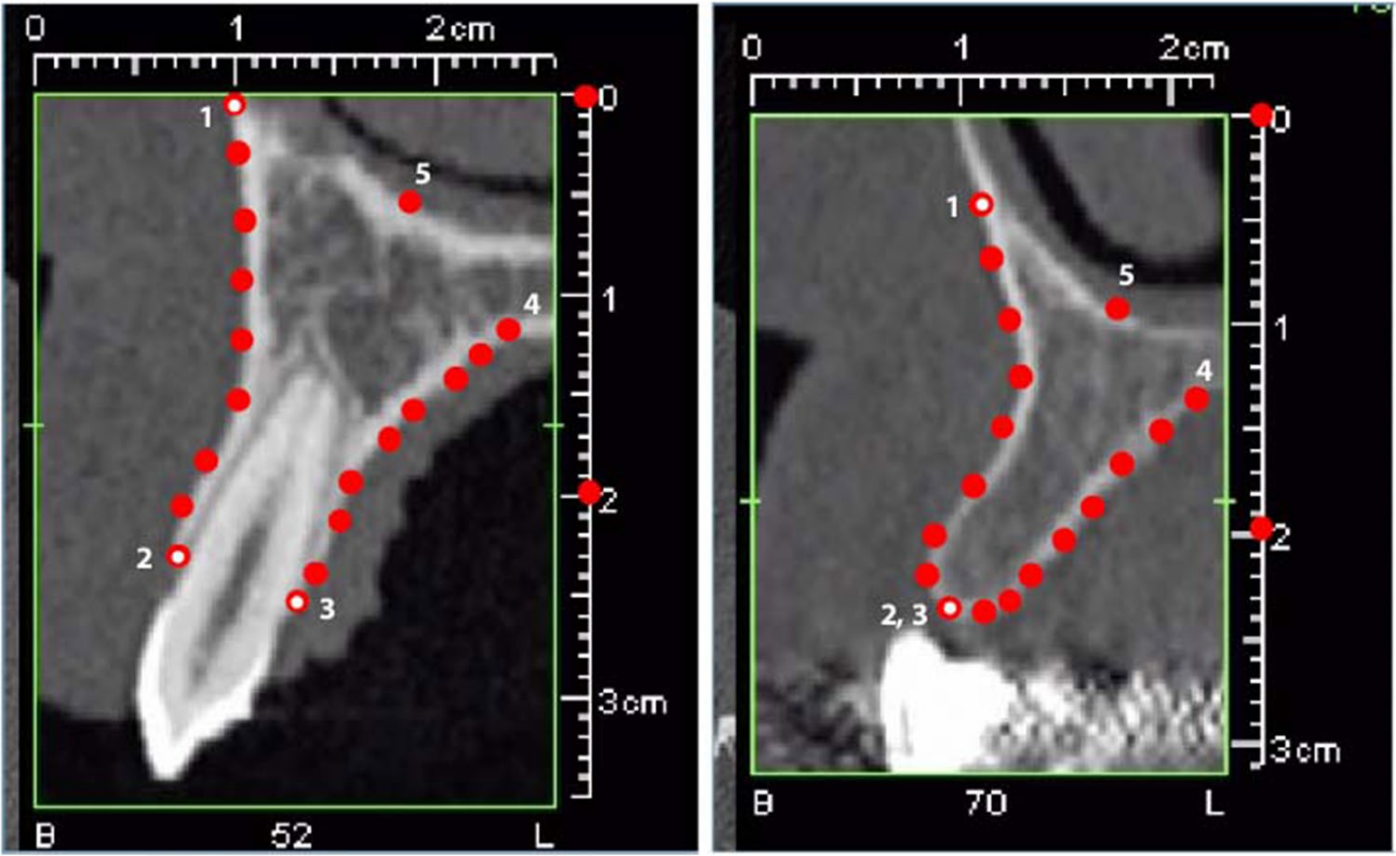
Landmark scheme used in this study, shown once on a cross-section with a lateral incisor in situ (left) and once in a case of agenesis. Landmarks 1, 2, and 3 are anatomical landmarks; the remaining 16 landmarks (red dots) are semilandmarks. Landmarks 2 and 3 (buccal and palatal alveolar crest) are placed at the same location on the alveolar ridge when no tooth was in situ

All images were arranged with the buccal side to the left and the palatal side to the right and presented in a random order to a single investigator (L.S.). For each individual, one cross-sectional picture was selected at either the mesio-distal midpoint of the lateral incisor tooth crown (control group) or at the center of the gap between central incisor and canine (agenesis and extraction). All images were randomized, and the following landmarks were digitized (see Fig. 1): (landmark 1) the most ventral point of the buccal curvature, (lm. 2 and 3) two points on the buccal and palatal alveolar crest, (lm. 4) the deepest point on the contour of the palatal curvature, and (lm. 5) one central point on the nasal floor. Landmarks 2 and 3 were placed at the same location on the alveolar ridge when no tooth was in situ. Additionally, 14 semi-landmarks were placed approximately equidistantly along the buccal contour of the maxilla and the contour of the palate (lm. 6–19). The landmark configurations were scaled based on a measured scale bar, and oriented along the central axis of the alveolar ridge by projecting the landmarks of each configuration onto the two principal components of landmarks 1–5. Randomization of images and location of landmarks were performed using tpsUtil (version 1.68) and tpsDIG2 (version 2.22; James Rohlf). To assess intra-rater reliability, 33 images of the agenesis group were measured twice in a random order.

### Morphometric analysis

In total, the sample comprised 161 configurations (including the contralateral sides) of 19 landmarks each. The sample consists of seven subsets: unilateral agenesis (*A*_uni_, *N* = 12), bilateral agenesis (*A*_bi_, *N* = 25), tooth extraction (Ex, *N* = 24), control group with the incisors in place (CG, *N* = 40), contralateral side of *A*_uni_ (*A*_uni_Cont, *N* = 13), contralateral side of *A*_bi_ (*A*_bi_Cont, *N* = 25), and contralateral side of Ex (ExCont, *N* = 20). The deviating sample sizes of the contralateral sides result from cases that had to be excluded because of image quality. Only landmarks 1, 2, and 3 were treated as fixed anatomical landmarks; all other 16 landmarks were treated as semilandmarks and their positions along the bone outline were estimated using the sliding landmark algorithm [43, 44] (Fig. 1). This involves an iterative sliding of the semilandmarks along their outline curves so as to minimize the Procrustes distance, a measure of overall shape difference, between each configuration and the sample mean shape. The more common approach to minimize bending energy was not applicable here because the large-scale component of shape variation was not sufficiently constrained by the three anatomical landmarks. Thereafter, all configurations were superimposed by generalized Procrustes analysis [45, 46] in order to standardize for variation in overall location, scale, and orientation. Based on the resulting sets of Procrustes shape coordinates, group mean shapes were computed and analyzed by principal component analysis (PCA). Shape differences were visualized by series of reconstructed shapes [39, 47]. Additionally, a between-group PCA of all individuals was performed [48]. Type I errors for multivariate differences in group mean shapes were estimated using permutation tests with 5000 random permutations and the Procrustes distance between the mean shapes as test statistic [41, 49].

In addition to shape, we also studied the size of the maxillary cross-sections. But because the length and width of the alveolar ridge differed considerable in their statistical behavior and also in their functional relevance, the variation in the vertical and horizontal dimensions of the maxillary cross-sections was studied separately. To this end, the centroid size (square root of the summed squared differences from the mean value) was computed separately for the x and y coordinates of each configuration. This allowed us to quantify variation in the overall length and width of the alveolar ridge, without referring to any single distance measurement. Type I errors for differences in the vertical and horizontal dimensions were estimated by one-way ANOVA (histograms indicated no major deviations from normality). All morphometric and statistical analyses were performed in Mathematica 12 (Wolfram Research Inc.).

Intra-rater reliability was estimated by the intraclass correlation coefficients (ICC) applied to the first two principal components as well as to the vertical and horizontal dimensions of the maxillary cross-sections.

## Results

**Fig. 2:**
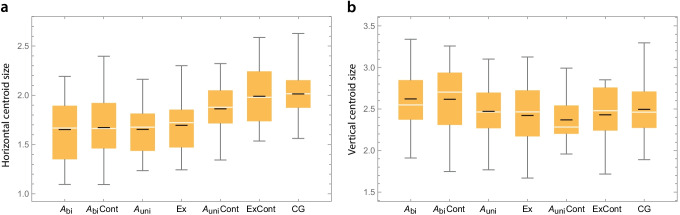
Box plots showing the distribution of overall width (**A**) and length (**B**) of the alveolar process at the upper lateral incisor in the seven groups. (*A*_bi_ = bilateral agenesis; *A*_bi_Cont = contralateral side of *A*_bi_; *A*_uni_ = unilateral agenesis; Ex = tooth extraction; *A*_uni_Cont = contralateral side of *A*_uni_; ExCont = contralateral side of Ex; CG = control group). The black lines represent the group mean for each group and the white lines the median

We plotted the vertical and horizontal dimensions for the following seven subsets of the sample: unilateral agenesis (*A*_uni_, *N* = 12), bilateral agenesis (*A*_bi_, *N* = 25), tooth extraction (Ex, *N* = 24), control group with the incisors in place (CG, *N* = 40), contralateral side of *A*_uni_ (*A*_uni_Cont, *N* = 13), contralateral side of *A*_bi_ (*A*_bi_Cont, *N* = 25), and contralateral side of Ex (ExCont, *N* = 20). The overall width of the alveolar ridge differed significantly among the seven groups (one-way ANOVA, *F* = 8.87, *p* < 0.001), where the three agenesis groups (*A*_uni_, *A*_bi_, *A*_bi_Cont) and the group with the extracted incisor had the narrowest alveolar ridge. The control group and the contralateral side of the extracted tooth had the widest alveolar dimensions, and the contralateral side of the unilateral agenesis had an intermediate width (Fig. 2A). In fact, the alveolar ridge was significantly narrower in *A*_uni_Cont than in the control group (*p* = 0.042 despite the small size of the *A*_uni_Cont sample), even though in both groups the incisor was in situ. The height of the alveolar ridge did not significantly differ among the seven groups (ANOVA, *F* = 1.61, *p* = 0.15) and showed no systematic relationship with agenesis (Fig. 2B).

**Fig. 3:**
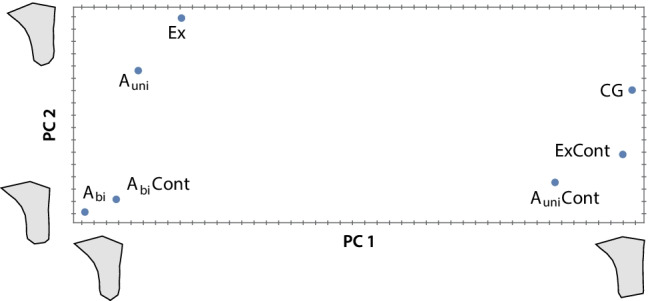
The first two principal components (PCs) of the group mean shapes (group names as in Fig. 2). The maxillary shapes corresponding to the extreme values along PC 1 and PC 2 are visualized by the gray polygons

**Fig. 4:**
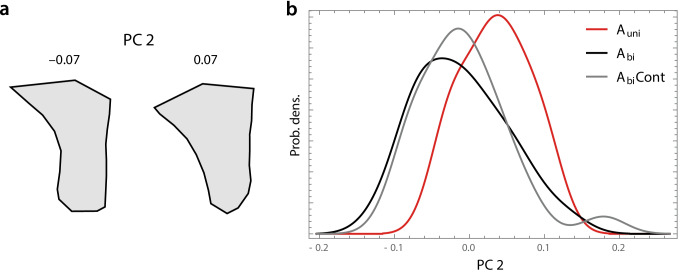
**A** Twofold extrapolation of the shape variation along PC 2 as depicted in Fig. 3, which reflects the ventral curvature of the maxillary alveolar ridge. These two shapes correspond to PC 2 scores of − 0.07 and 0.07, respectively. **B** Smooth histograms of the PC 2 scores for the unilateral agenesis group (red) and the two bilateral agenesis groups (black, gray)

Figure 3 shows the first two principal components (PCs) of the group mean shapes of the seven groups, accounting for 93.6% of total shape variation. PC 1 corresponds to the relative width of the alveolar ridge and separates the groups with an incisor in situ (high PC scores) from those with a missing incisor (low scores). The ordination of the groups along PC1 closely resembles that of the average horizontal dimensions in Fig. 2A. PC 2 reflects the ventral curvature of the maxillary alveolar ridge (Fig. 3, Fig. 4A) and primarily separates the two bilateral agenesis groups from those with unilateral agenesis and unilateral tooth extraction. In the bilateral agenesis group, this curvature was more pronounced than in the unilateral agenesis and tooth extraction groups. Among the groups with an incisor in situ, the contralateral side of unilateral agenesis (*A*_uni_Cont) had the lowest PC 2 scores, i.e., the most pronounced ventral curvature.

Individual variation within groups was relatively large (Suppl. Figure 1), but the unilateral and bilateral agenesis groups differed clearly in their mean score along PC 2 (Fig. 4B). Because of the small unilateral agenesis sample (*N* = 12), the mean differences between bilateral and unilateral agenesis groups were at the border of statistical significance (*A*_uni_ vs. *A*_bi_ *p* = 0.08, *A*_uni_ vs. *A*_bi_Cont *p* = 0.12). However, the mean shapes of both bilateral agenesis groups differed significantly from the unilateral extraction group (*p* = 0.008, *p* = 0.023).

Intraclass correlation coefficients were 0.96 and 0.88 for PC1 and PC 2, respectively. The vertical and horizontal dimensions both showed intraclass correlations of 0.93. This indicates a relatively small measurement error and high intra-rater reliability. But as these coefficients were calculated from individuals of the agenesis group only, intraclass correlations among groups is likely even higher than within group.

## Discussion

In this retrospective study, we compared the size and shape of the maxillary alveolar ridge in patients with bilateral or unilateral agenesis of the lateral incisor to patients with an extracted lateral incisor and patients with all lateral incisors in situ. We found that both the absolute dimensions of the alveolar ridge as well as its cross-sectional shape vary strongly among individuals. The corresponding statistical distributions overlapped among all groups. However, the average alveolar width (PC1) was clearly larger in the dentulous groups compared to patients with agenesis or extracted lateral incisors. Among edentulous groups, the shape of the ventral curvature of the alveolar ridge (PC 2) differed on average between bilateral agenesis and unilateral agenesis as well as post tooth extraction: in the bilateral agenesis group the buccal curvature of the alveolar ridge was most pronounced.

Alveolar ridge formation is strongly affected by tooth development and eruption [50]. In patients with agenesis of the upper lateral incisors, a labial concavity of the alveolar ridge is frequently present [51, 52]. Our results show a more pronounced ventral curvature of the alveolar ridge in individuals with bilateral lateral incisor agenesis in comparison to those with unilateral agenesis. This fits with the well-documented association of alveolar cross-sectional form and tooth agenesis. It also appears to indicate a positive correlation between the extents of these anomalies. Similar findings were reported for other teeth [53–56]. For instance, a previous study on lower second premolar agenesis revealed that cross-sectional mandibular size and shape significantly differs among individuals with and without agenesis of the lower second premolars [20].

Among our edentulous groups, the ventral curvature appeared least pronounced in patients, where the lateral incisor had been present but extracted. It is well documented that the residual alveolar ridge undergoes marked dimensional alterations after tooth extraction [57]: up to 35% bucco-oral width reduction within the first three months has been reported [58]. However, this reduction primarily occurs at the edge of the alveolar ridge, with a bucco-palatal width reduction resulting in a knife-edged shape rather than an accentuation of the ventral curvature.

Our first hypothesis, namely that the developing tooth affects maxillary growth, is clearly verified. Interestingly, we found that alveolar ridge shape differs on average between bilateral agenesis and the affected side of unilateral agenesis, even though in both cases an incisor is absent. Similarly, we found that the alveolar width of the unaffected side of unilateral agenesis tends to be smaller than in the control group (*p* = 0.042 despite the small size of the *A*_uni_Cont group), even though in both cases the incisor is in situ. Both findings cannot be explained by H1. Instead, they suggest that maxillary shape has an effect on dental agenesis (H2) or that both are influenced by a shared common factor (H3), e.g., some pleiotropic genes. Potentially, even all three causal pathways (H1–H3) could be present.

Without an experimental design, it is difficult to distinguish between H2 and H3 based on our morphometric data, but the overlap of the groups in their size and shape distributions does not support a universal threshold of alveolar width necessary for dental development. However, such a threshold could depend on dental and cranial sizes, among other factors, and thus vary among individuals. From a statistical perspective, the relationship appears like a probabilistic one: The narrower the alveolar ridge and the more concavely shaped its ventral curvature, the more likely is the agenesis of the lateral incisor.

Clinically, the treatment of a missing lateral incisor is dictated by the width of the alveolar ridge. It has been shown that straightforward implant placement in this region has a tendency to be less likely if the tooth is missing due to agenesis rather than extraction, i.e., the more pronounced buccal curvature of the alveolar ridge might result in a higher need of bone augmentation prior to or in combination with implant placement than the crestal width. The present results suggest that this effect may be aggravated in cases of bilateral agenesis.

Some limitations — primarily due to the retrospective design — should be considered. Several parameters that potentially affect post-extraction bone remodeling were not completely available for all patients and thus not considered in the analysis, for example, presence of systemic diseases and medication, smoking status, and the exact timepoint of tooth extraction. Nevertheless, the presence of any augmentation material was defined as exclusion criterion and a minimum of 3 months passed between tooth extraction and CT recording. Another potential bias in the agenesis group is orthodontic treatment prior to CT scanning. This information was available for 19 patients, of which 12 patients received treatment, including space opening in the region of the missing lateral incisor. However — as reported previously [14] — none of the parameters defining the alveolar ridge dimension (i.e., bucco-palatal width, area, and height) presented any significant differences between these two groups (i.e., space opening vs. no space opening).

## Conclusions

In summary, we found an association of the maxillary alveolar ridge and agenesis of the lateral incisor, but this association is not only a simple, unidirectional result of the absent permanent tooth. Instead, a too small or inappropriately shaped alveolar ridge may also inhibit normal formation or development of the tooth bud, thus leading to dental agenesis in this anatomical region. It is also possible that pleiotropic genetic and developmental factors influence both maxillary growth and dental development. Presumably, all three causal pathways contribute to the observed association.

## Supplementary Information

Below is the link to the electronic supplementary material.

Supplementary file1 (PDF 396 KB)

## Data Availability

The data supporting the findings of this article are openly available in the Open Science Framework (OSF) repository at: https://osf.io/72kxc/.
