# Supplementary figures and images for: Maxillary lateral incisor agenesis is associated with maxillary form: a geometric morphometric analysis
Source: Clin Oral Investig. 2022 Aug 29;27(3):1063–70. doi: 10.1007/s00784-022-04690-9 (PMC9985555; doi:10.1007/s00784-022-04690-9)

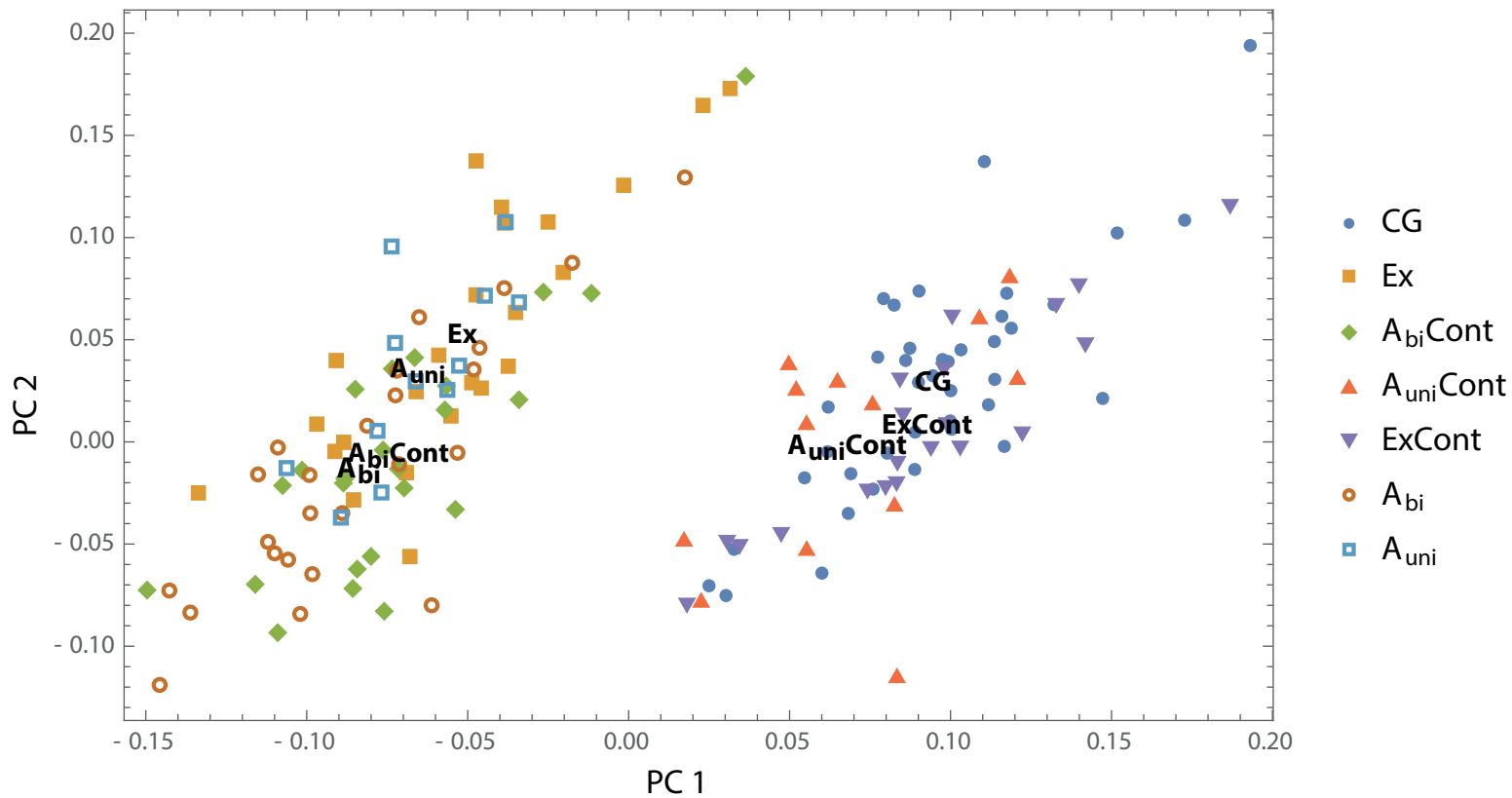

Supplement: Supplementary file 1 — Supplementary file1 (PDF 396 KB) [file 784_2022_4690_MOESM1_ESM.pdf]
